# Structural dynamics of the transaminase active site revealed by the crystal structure of a co-factor free omega-transaminase from *Vibrio fluvialis* JS17

**DOI:** 10.1038/s41598-018-29846-0

**Published:** 2018-07-30

**Authors:** Young-Cheul Shin, Hyungdon Yun, Hyun Ho Park

**Affiliations:** 1 Department of Cell Biology, Harvard Medical School, Boston, Massachusetts 02115 USA; 2 Department of Bioscience & Biotechnology, Konkuk University, Seoul, 143-701 Republic of Korea; 3 College of Pharmacy, Chung-Ang University, Seoul, 06974 Republic of Korea

## Abstract

Omega (ω)-transaminase catalyzes the transfer of an amino group from a non-α position amino acid, or an amine compound with no carboxylic group, to an amino acceptor, and has been studied intensively because of its high potential utility in industry and pharmatheutics. The ω-transaminase from *Vibrio fluvialis JS17 (Vfat)* is an amine:pyruvate transaminase capable of the stereo-selective transamination of arylic chiral amines. This enzyme exhibits extraordinary enantio-selectivity, and has a rapid reaction rate for chiral amine substrates. In this study, we report the crystal structure of the apo form of *Vfat*. The overall structure of *Vfat* was typical of other class III aminotransferase exhibiting an N-terminal helical domain, a small domain, and a large domain. Interestingly, the two subunits of apo *Vfat* in the asymmetric unit had different structures. A comparison of the overall structure to other transaminases, revealed that the structures of the N-terminal helical domain and the large domain can be affected by cofactor occupancy, but the structural rearrangement in these regions can occur independently.

## Introduction

Transaminases (also called aminotransferases) are a large group of enzymes that can remove the amino group from amino acids, transferring it to an α-keto acid, thereby producing an amino acid product. Transaminases therefore play an important role in amino-acid metabolism^1,2^. All transaminases use pyridoxal 5ʹ-phosphate (PLP) as a cofactor for catalysis. The catalytic mechanism of a transaminase can be divided into two steps; first the PLP cofactor changes from the aldehyde form (PLP) to the amino form (PMP). This is accomplished by the amino acid substrate donating its amine group to the PLP cofactor in the enzyme to produce E-PMP and the corresponding keto acid. In the second part of the reaction, the amino acceptor takes the amino group from E-PMP to produce the corresponding amino acid, thereby regenerating E-PLP^3,4^.

Transaminases can be classified into α- or ω-transaminase based on the relative position of the amino group to be transferred^5,6^. The family of ω-transaminase, which use ω-amino acids, include pyruvate transaminase, ornithine transaminase, 4-aminobutyrate transaminase, and acetylornithine transaminase, all of which transfer amino groups on non-α positions^7,8^. On the other hand, α-transaminase act only on the α-amino groups on α-amino acids. Based on sequence similarity, transaminases can also be divided into four subgroups and ω-transaminase belongs to subgroup II^2^. Among the ω-transaminases, only ω-amino acid:pyruvate transaminase has a catalytic activity towards primary amines and aliphatic amines that lack carboxyl groups^6,9,10^. ω-transaminases are particularly interesting and have been studied intensively because of their industrial application as biocatalysts for the production of various amino acids and chiral amines of high enantioselective purity^11–14^.

The ω-transaminase from *Vibrio fluvialis* JS17 (Vfat) is an unique PLP-containing enzyme that catalyzes the reversible amino group transfer reaction from an amine to a keto acid^10^. Vfat is an amine:pyruvate transaminase capable of the stereo-selective transamination of arylic chiral amines that is highly suitable for the production of chiral amines due to its strict enantio-selectivity and its broad specificity for chiral amine amino donors^10^. Interestingly, Vfat showed narrow substrate specificity toward aliphatic amines, although it has high substrate specificity for chiral aromatic amines and enantio-selectivity for the (S)-enantiomer of chiral amines^3^. To improve the activity and substrate specificity of Vfat, rational protein design by modeling and direct enzyme evolution have been attempted^15–20^. In the present study, we report the crystal structure of the PLP free apo Vfat. Although the overall structure of the cofactor free Vfat was similar to that of the recently solved PLP-bound Vfat^17^, the two subunits of the cofactor free Vfat in the asymmetric unit showed different structures. Based on the current structural study, we found that the structures of the N-terminal helical domain and the large domain of Vfat can be altered by cofactor occupancy in the PLP binding site. In addition, we discovered that the two regional rearrangements of the structure that occur upon cofactor binding occur independently. A model of activation and inactivation of transaminase by PLP-substrate dissociation, enzyme stability and stoichiometric changes has been suggested recently^21–23^. Our findings with structure of dimeric, PLP-free form of apo Vfat provide a better understanding of structural alternation and stability of transaminase without cofactor

## Results

### Structure of the apo state of ω-transaminase from *Vibrio fluvialis* JS17 (Vfat)

**Figure 1:**
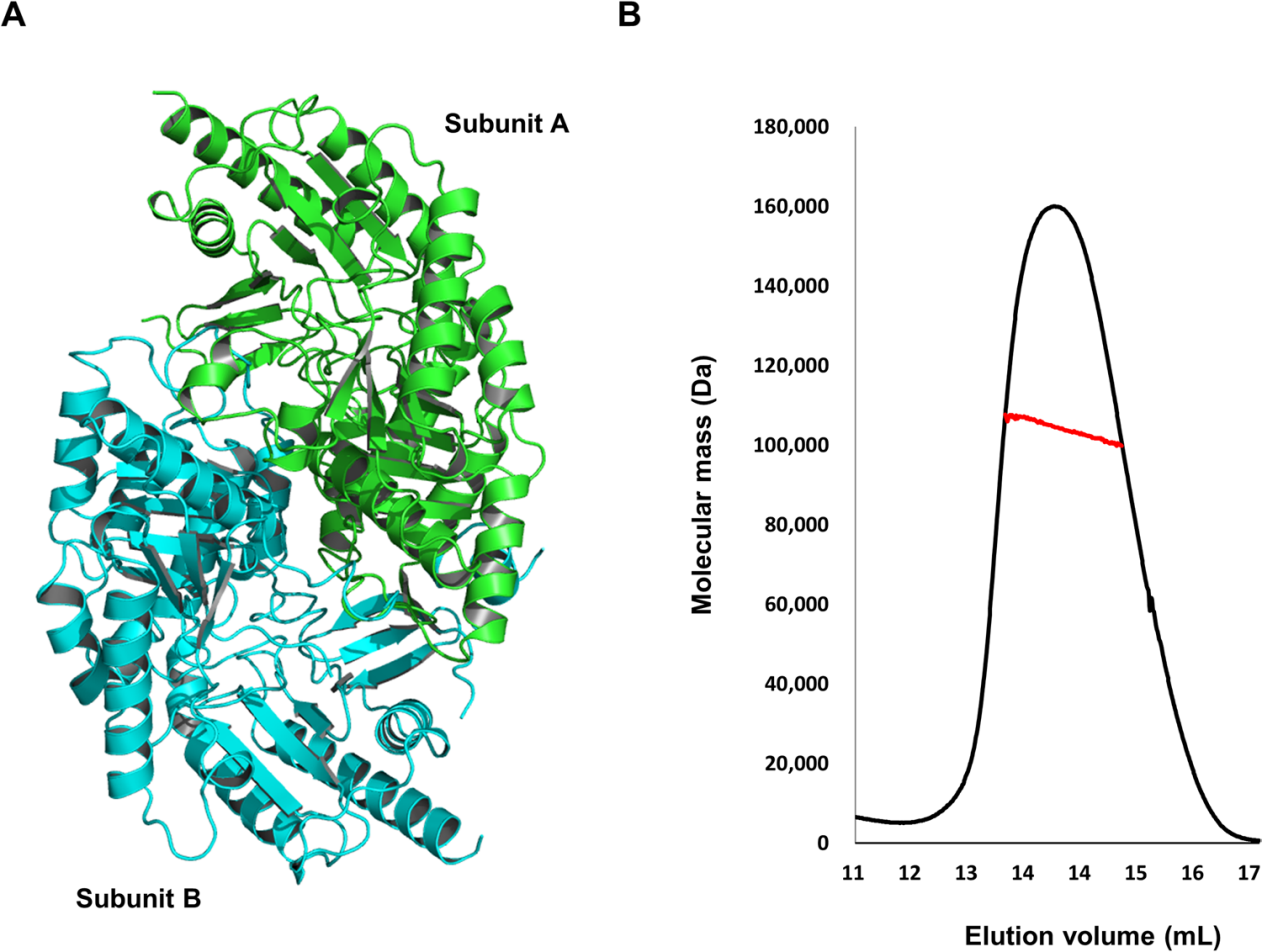
Dimeric structure of ω-transaminase from *Vibrio fluvialis* JS17 (Vfat). (**A**) Overall dimeric structure of Vfat. The individual subunits are shown in different colors. (**B**) MALS data. The red line indicates the experimental data.

The crystal structure of ω-transaminase from *Vibrio fluvialis* JS17 (Vfat) was determined at a resolution of 2.0 Å using the coordinates of the previously solved Class III aminotransferase (PDB ID: 3GJU)^24^ as the search model for molecular replacement. The structure was refined to an Rwork = 18.7% and Rfree = 23.7%. The crystallographic and refinement statistics are summarized in Table 1. Several residues with poor electron density were excluded from the final model. Two molecules, subunit A and subunit B, were detected in the asymmetric unit (Fig. 1A). Interestingly, the two subunits did not contain any cofactor such as PMP or PLP based on the electron density map (Supplementary Fig. 1A,B). As the dimeric form of the current structure might be coincidently formed during crystal packing, we confirmed the stoichiometry of the apo-state of Vfat in solution by multi-angle light scattering (MALS), which measures the absolute molecular mass of the target protein in solution. The theoretical molecular mass of Vfat, including the His-tag was 52.79 kDa, whereas the measured mass was 103.72 kDa (0.89% fitting error), indicating that the apo-state of Vfat also forms a dimer in solution (Fig. 1B).

**Table 1:** Crystallographic statistics.

Data collection	Native
Space group	*P2*_1_2_1_2_1_
Cell dimensions	
*a*, *b*, *c*	78.28 Å, 95.31 Å, 123.03 Å
Resolution	75–2.0 Å
^†^ *R*_sym_	9.1% (48.0%)
^†^*I*/σ*I*	41.6 (4.2)
^†^Completeness	99.8% (99.8%)
^†^Redundancy	6.8 (6.8)
**Refinement**	
Resolution	48.33–2.0 Å
No. reflections used (completeness)	59,798 (99.7%)
*R*_work_/*R*_free_	18.7. %/23.7%
No. atoms	
Protein	6,689
Water and other small molecules	615
Average B-factors	
Protein	24.5 Å^2^
Water and other small molecules	23.4 Å^2^
r.m.s deviations	
Bond lengths	0.019 Å
Bond angles	1.923°
Ramachandran Plot	
Most favored regions	95.1%
Additional allowed regions	4.2%

^†^The highest resolution shells are shown in parentheses.

**Figure 2:**
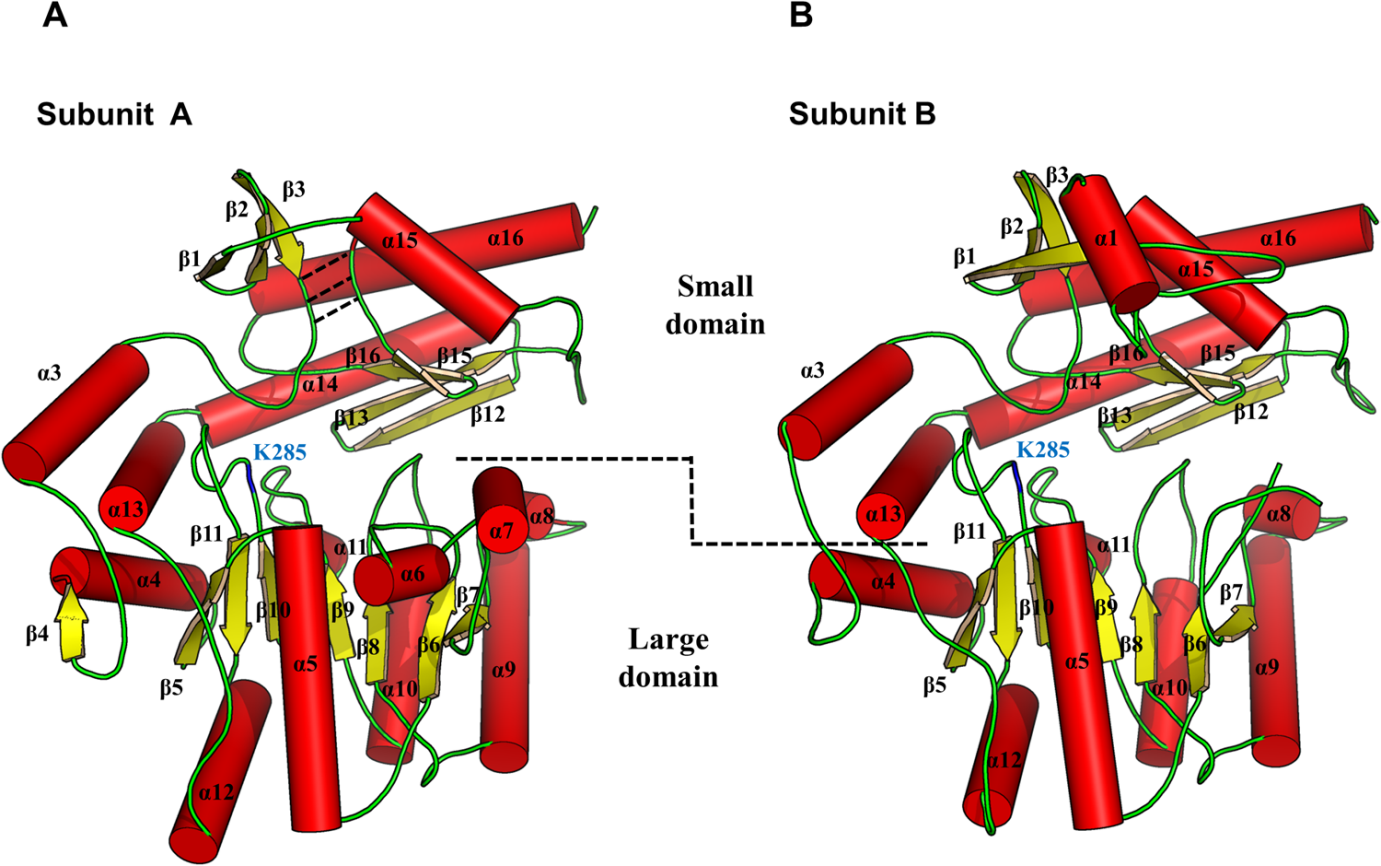
Folding analysis of the apo-state of Vfat subunit. The α-helices and the β-strands are represented as red cylinders and yellow arrows, respectively. Loops are colored in green. Lys285 (K285) is represented as a blue loop. (**A**) The N-terminal lobe and C-terminal lobe in the small domain are connected laterally by hydrogen bonds, represented by the dashed line. In the final model, the α1–2 helix domain is not visible due to poor electron density. (**B**) In contrast to subunit A, subunit B has an ordered α1 helix while α6–7 helix domain is disordered.

The overall structure of apo Vfat shows the typical transaminase fold, which is comprised of two major domains, the small domain and the large domain (Fig. 2A). The large domain (residues 91–313) forms a Rossmann-like fold, which is composed of a seven-stranded parallel β sheet (Fig. 2A). All the β strands except β11 were parallel with the strand order β5-β11-β10-β9-β8-β6-β7 (Fig. 2A,B). The small domain can be divided into two lobes, the N-terminal lobe (residues 31–79) and the C-terminal lobe (residues 327–452). The two lobes are connected laterally by three backbone hydrogen bonds formed between residues 50–52 from the N-terminal lobe and residues 411–413 from the C-terminal lobe (Fig. 2A). The N-terminal lobe in the small domain contains three-stranded antiparallel β strands which are capped by two helices, known as the additional domain α1-α2 helices (N-terminal helix domain). Interestingly, the electron density for this domain was poorly visible in the 2Fo-Fc electron density map. Residues 1–29 in subunit A were completely disordered (Fig. 2A), while residues 24–28 in subunit B were partially disordered (Fig. 2B and Supplementary Fig. 2A). An invisible region, arising as a result of poor density, was also observed in the α6-α7 helix domain of subunit B (corresponding residues 151–163). Thus, this region was not resolved in the final model (Fig. 2B and Supplementary Fig. 2B).

### Comparison with the cofactor-bound forms of Vfat

**Figure 3:**
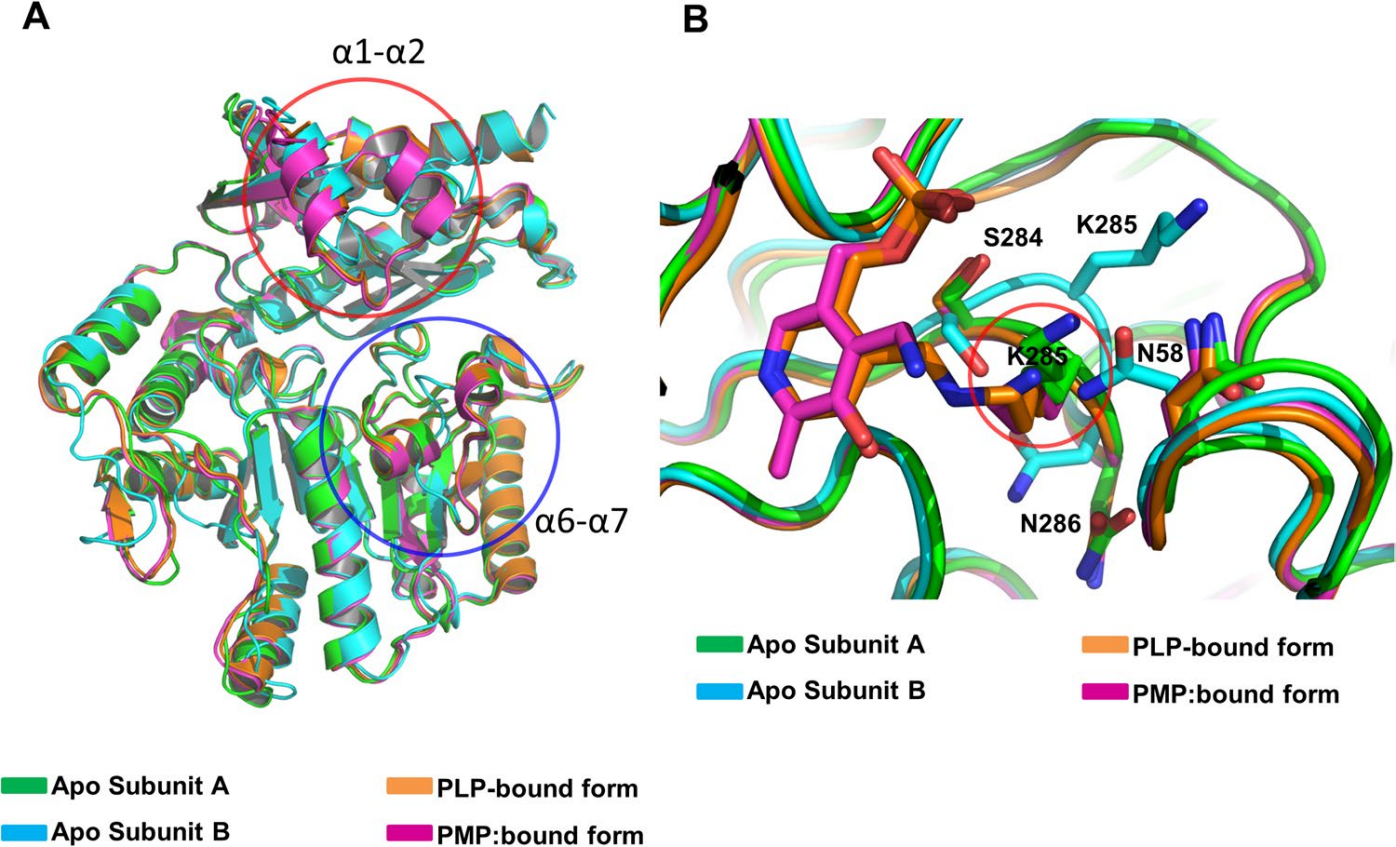
Structural comparison of PLP-bound, PMP-bound, and apo Vfat. (**A**) The structurally altered α1-α2 (Red circle) and α6-α7 (Blue circle) regions. (**B**) Structural comparison of the region that contains K285 residue at the active site. The red circle indicates the typical structure of K285 detected in the structure of PLP-bound and PMP bound Vfat.

As the structures of PLP/PMP bound Vfats, namely the PLP-bound form (PDB: 4E3R) and the PMP-bound form (PDB: 4E3Q), are available^17^, we compared our structure with PLP/PMP bound Vfats. The structure of the two PLP/PMP bound Vfats differed from our apo Vfat structure in that both of the two cofactor-bound forms (PLP/PMP) of Vfat had structurally stable helices in the α1-α2 region and the α6-α7 region (Fig. 3A) unlike the cofactor free Vfat form whose structure contained a disordered α1-α2 region in subunit A and a disordered α6-α7 in subunit B. The PLP/PMP bound Vfat had four symmetric molecules in the asymmetric unit^17^. Next, we compared the structural differences in the active site between PLP-bound form, the PMP-bound form and the cofactor-free apo form of Vfat. It is well known that the active site lysine for PLP binding is highly conserved among all transaminases. In Vfat, the cofactor can bind the lysine present at residue 285 (K285), which is located between β10 and β11 (Fig. 3B). In the PLP-bound form (PDB ID: 4E3R), PLP forms a covalent Schiff base linking it to K285. In the PMP-bound form (PDB ID: 4E3Q), K285 was not covalently bound to PMP, but the orientation of the side chain group of K285 in the active site was identical to the PLP-bound form (Fig. 3B). Interestingly, however, the two subunits in the apo form of Vfat showed different side chain orientation at K285 despite the backbone folding in the vicinity of the K285 in both subunits being structurally analogous to those of the PLP- and PMP-bound form. Specifically, the side chain group of K285 in subunit A showed a similar orientation to that of PMP-bound form, whereas the side chain group of K285 in subunit B had the opposite orientation (Fig. 3B). Generally, free residues that lose their binding moieties may be less structurally stable. In this regard we think that the absence of cofactor could lead to a loss of conformational stability of K285. Differences in secondary structure in several regions were also observed, and are summarized in Supplementary Fig. 3. A cofactor-induced structural change in the active site has also been reported in a previous structural study of the ω-transaminase from *Chromobacterium violaceum* (Cv-ωTA)^25^. According to this study, the structure of apo Cv-ωTA and PLP-bound Cv-ωTA were different and PLP binding induced a structural change in Cv-ωTA, which is similar to our observations in this study.

### Conformational diversity in the substrate pocket

**Figure 4:**
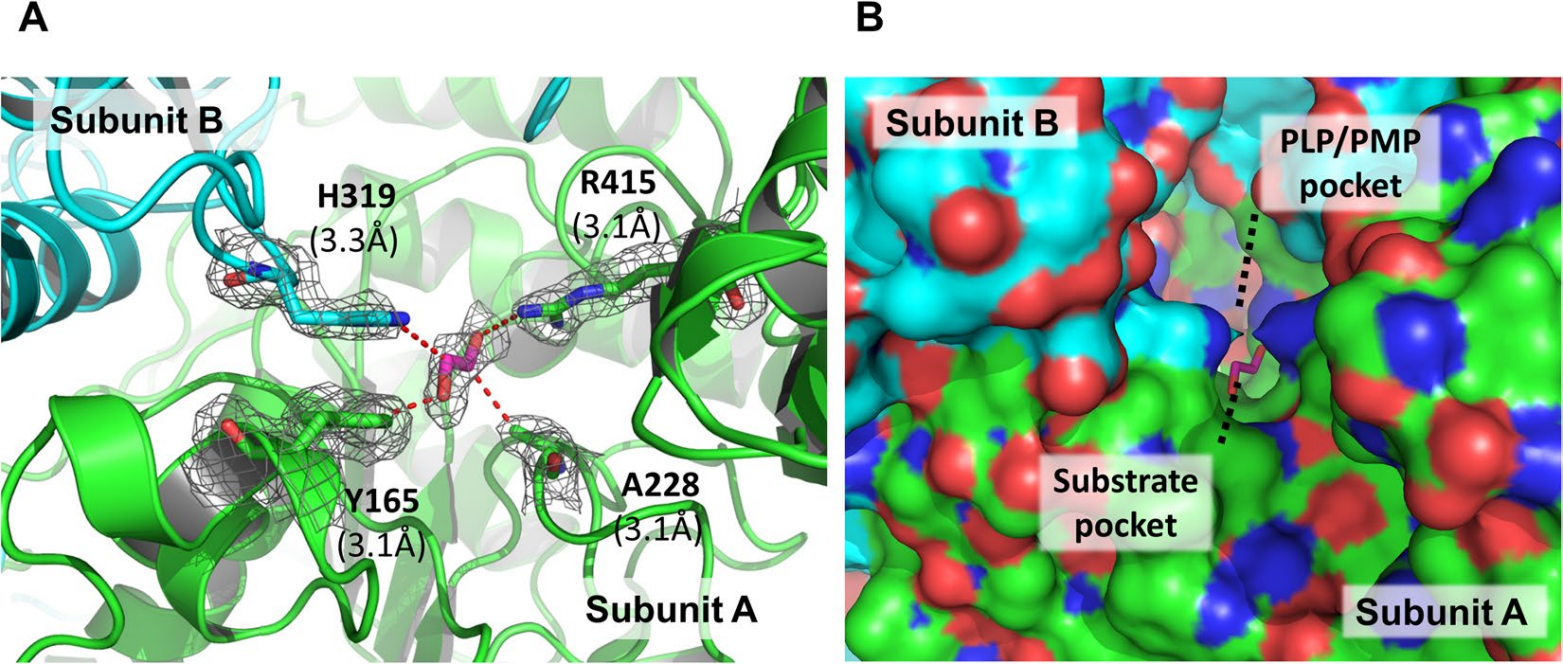
Substrate pocket in the A subunit of apo Vfat. (**A**) The substrate pocket in apo Vfat. The stick representation shows that four residues that are in close proximity (within 3.5 Å) from an unidentified, substrate-like, molecule. (**B**) Close-up surface representation of the cofactor- and ligand-binding site entrances in subunit A of apo Vfat. The unidentified, substrate-like, molecule is shown as a red-stick.

As mentioned above, the apo Vfat subunits are structurally different to the PLP/PMP-bound form of Vfat, especially in the catalytic region. Although the K285 is not bound to its cofactor, subunit A appeared to be stabilized in the catalytic region. In addition, the α6-α7 helix domain in subunit A of apo Vfat was also structurally stable, unlike subunit B, which led us to think that the two subunits were not in the same state. Thus, we further explored the possibility that a molecule may be occupying a space in the vicinity of the catalytic region in the two subunits of our apo Vfat. After careful analysis of the structure, and by lowering the contours of the Fo-Fc map, we discovered a small volume of electron density around the substrate pocket of subunit A. After several rounds of restrained refinement with Refmac5, we determined the electron density for the small molecule to be around the substrate pocket in subunit A (Fig. 4A). Four residues (Y165, A228, and R415 from subunit A, and H319 from subunit B) were found within 3.5 Å of this electron density and form a small inside cavity which is similar to the binding pocket for substrates in other transaminases (Fig. 4B). The size and shape of this unidentified density is similar with ethylene glycol. However, since no ethylene glycol was added in the crystallization buffer, it might be an impurity. To our knowledge, however, this pocket seems to be unnatural because the volume was significantly smaller than the general substrate pocket which has been observed in other transaminases^12,25,26^. In addition, a small molecule in the apo state of Cv-ωTA was not found in the substrate pocket^25^. In contrast to subunit A, the electron density of this molecule and the related substrate pocket were not observed in the corresponding region of subunit B. Our observations suggest that the conformational diversities between subunit A and B in our apo Vfat structure are due to the presence of an unidentified small molecule in the substrate pocket.

### Rearranged loops at the dimer interface

**Figure 5:**
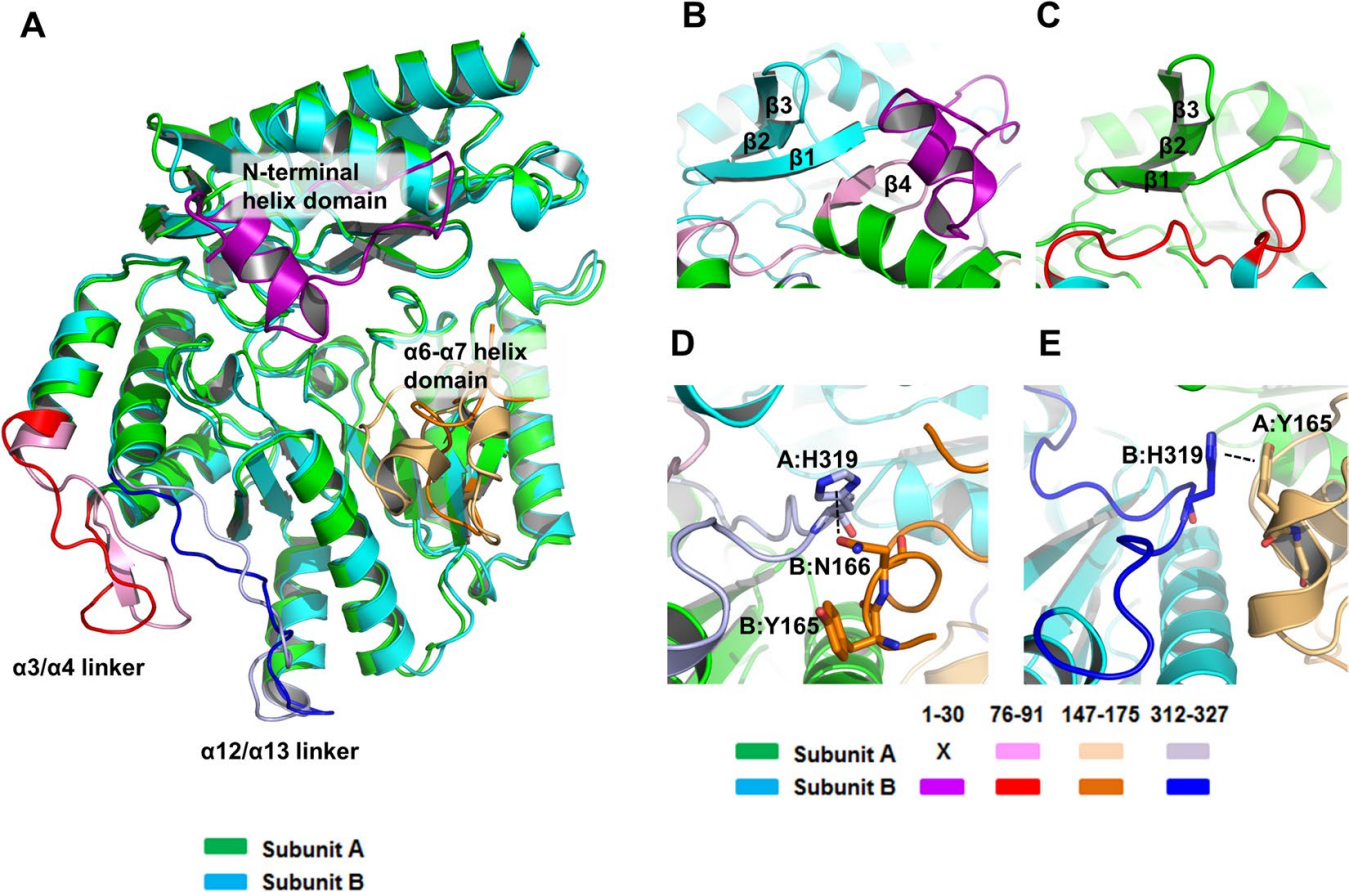
Comparison of subunit A and B in apo Vfat. (**A**) Superimposition of the structures of subunit A (green) and B (cyan) of apo Vfat revealed that α3/α4 linker loop (pink for subunit A and red for subunit B) and α12/α13 linker loop (light blue for subunit A and blue for subunit B) have significantly different conformations. The N-terminal helix domain (purple for subunit B) was disordered in subunit (**A**) In contrast to N-terminal helix domain, the α6-α7 helix domain was disordered in subunit B (light orange for subunit A and orange for subunit B). (**B**,**C**) Close-up presentation of the dimer interface in the vicinity of the α3/α4 linker loop from subunit A (pink color) (**B**) and B (red color) (**C**). The pink arrow indicates the β4 strand from subunit (**A**) In the N-terminal β1,2,3 sheet region, subunit A (green) was more wide than that of subunit B (cyan). (**D**) Interaction between H319 in subunit A and N166 in subunit. (**B**) The imidazole ring of H319 interacts with the delta oxygen on N166 in subunit B through an aromatic n → π* interaction. The α6/α7 helix from subunit B is colored orange. (**E**) Interaction between Y165 from subunit A and H319 from subunit B through π-stacking interactions.

**Figure 6:**
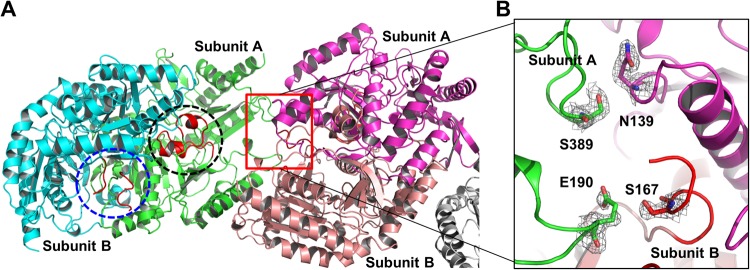
The position of α6/α7 helix that contains Y165 and N166. (**A**) The structure of dimeric apo Vfat (green and cyan color) with one of the neighboring molecules in crystal packing (magenta and pink color). The α6/α7 helix regions that contains Y165 and N166 are boxed with black-dotted circle (subunit A) and a blue-dotted circle (subunit B). (**B**) The crystal packing interface formed by subunit A and the unwound α6/α7 helix domain in subunit B.

The overall structure of the two subunits in apo Vfat were quite different, having a root mean square deviation (RMSD) of 2.04 Å. In particular, the active sites of the two subunits were different. Structural differences between the four types of Vfat (the PLP-bound form, the PMP-bound form, and the two subunits in the apo dimer) were also observed, especially around the α1-α2 and α6-α7 helix domains. To obtain further insight into this structural diversity, we further compared the structures of the four types of Vfat. Apart from the disordered regions in the apo Vfat dimer (1–29 in subunit A and 151–163 in subunit B), another two regions showed distinctly different conformations. These two regions were the linker loops which link two helices and are located on the interaction interface between two subunits (the dimer interface). One linker is at residues 78–91, which links α3 helix to α4 helix (α3/α4 linker). It contains a β strand (β4 at residues 88–90) from subunit A (colored pink in Fig. 5A), while the secondary structure of a β strand was not observed in the corresponding region in subunit B (colored red). In subunit A, the β4 strand was connected laterally by backbone hydrogen bonds with the β1 strand from subunit B, forming a four-stranded β-sheet (Fig. 5B). This connection was also observed in both PMP- and PMP bound Vfats^17^. In contrast, the conformation of the α3/α4 linker in subunit B differed from subunit A, and the secondary structure of the three residues (88–90) was observed as a loop (Fig. 5C). In addition, a shortened β1 strand structure was observed in subunit A compared with subunit B or the cofactor bound Vfats. It is concluded that the change of local motion of the α3/α4 linker in subunit B, structurally affected by the absence of PLP in the catalytic region, sterically interferes with the proper localization of the β1 strand in subunit A resulting in a shortening of the β1 strand secondary structure in subunit A (Supplementary Fig. 4). The change of local motion of the loop and subsequent loss of the structure were also observed at the dimer interface. The linker loop at residues 312–327, which links α12 helix to α13 helix (α12/α13 linker), had different conformations in subunit A and B (Fig. 5A). In subunit A, the α12/α13 linker loop (colored light blue) was shifted to the outside region, away from the center of monomer unit, and H319 interacted with N166 in subunit B through an aromatic n → π* interaction (Fig. 5D). In contrast, H319 in subunit B (colored blue) did not interact with N166 in subunit A but instead interacted with Y165 through a π-stacking interaction (Fig. 5E). In subunit A, Y165 and N166 were part of the α6/α7 helix, whereas the α6/α7 helix domain in subunit B was an unwound helix which affected crystal packing (Fig. 6A,B), differing from the symmetric tetramer unit of the PLP-and PMP-bound forms. Specifically, these interactions were not observed in either of the PLP- and PMP-bound forms of Vfat. Interestingly, Y165 in subunit A and H319 in subunit B in apo Vfat participated in the formation of the substrate pocket (Fig. 4A). Therefore, rearrangements in the dimer interface of apo Vfat might be affected by small molecules, because all subunits of PLP/PMP Vfats, which did not contain any molecule in the substrate pocket, had identical conformations in this region.

## Discussion

Our structural study showed that subunit A had structurally stable helices (α6 to α7) in the large domain and a disordered structure for residues 1–29 (α1 to α2). In contrast, subunit B had structurally unstable helices in the α6 to α7 region, while the α1 helix (residues 7–14) had stable folding unlike subunit A. In addition, rearranged loops were observed at the dimer interface, and these regions were not symmetrical between subunit A and B. These findings were not observed in the PLP- or PMP bound forms of Vfat, indicating that the structural diversity in the active site and dimer interface is a unique feature of cofactor free Vfat. When our Vfat was initially purified, the protein contained PLP or PMP. However, the cofactor was absent from the final structure, indicating that the cofactor was released from the protein during purification and crystallization steps by unknown reason. In this current dimeric apo Vfat structure that contains an unidentified small molecule in subunit A, we observed that the linker loops in the dimer interface and the catalytic region in subunit A was stabilized by small molecule binding. In addition, we also detected that the catalytic region in subunit B, which was affected by rearrangement of the adjacent region in subunit A, became extended and the conformational stability of several regions, including the α6-α7 helix domain in subunit B, decreased because of the loss of interaction.

**Figure 7:**
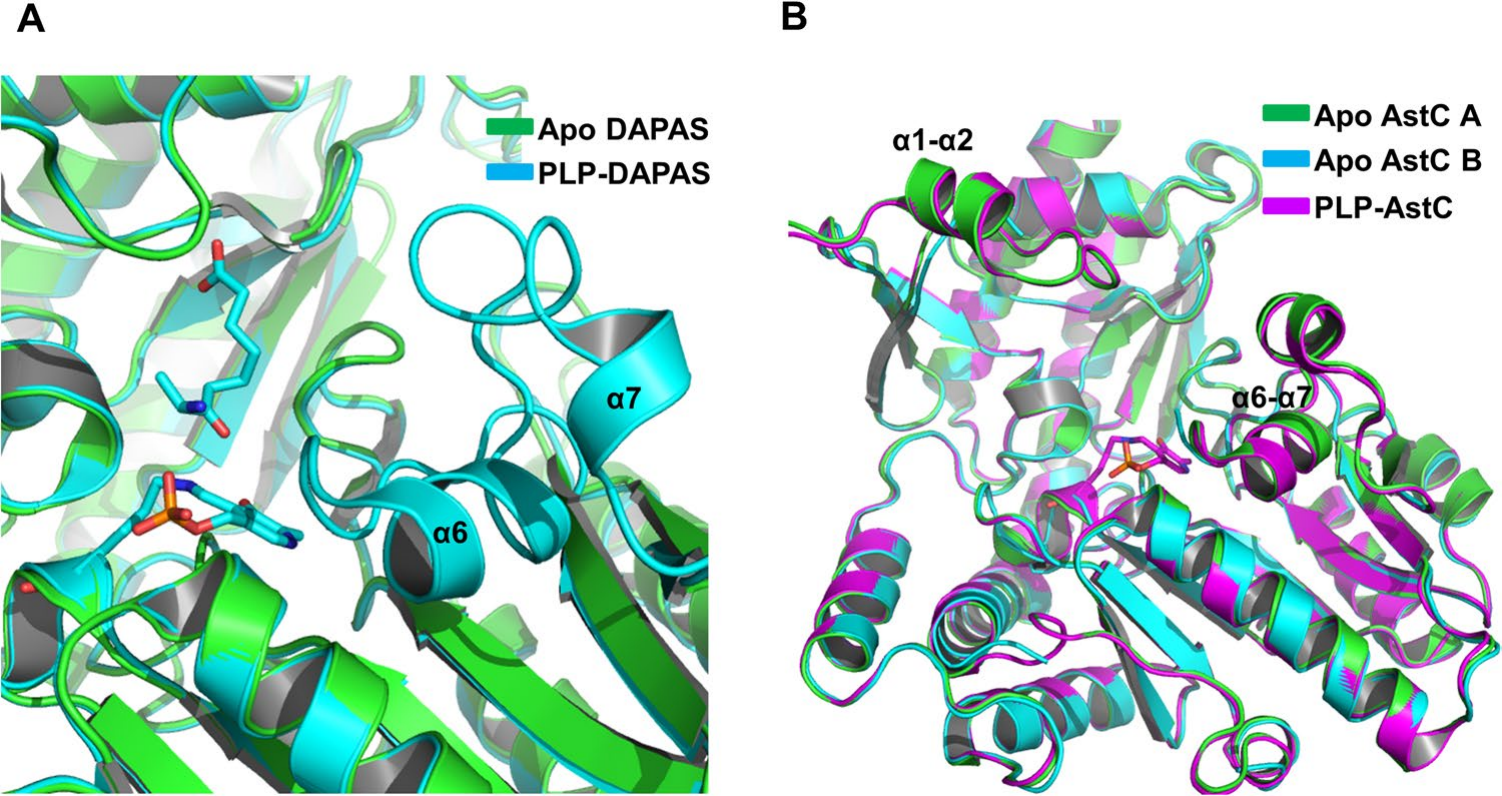
Similar structural diversity of the active site of other transaminases. (**A**) The structures of apo and PLP-bound Mtb DAPAS (PDB ID: 3DRD). The lost α6-α7 helix domain (residues at 144–172) is shown. (**B**) The structure of Apo and PLP-bound catabolic N-succinylornithine transaminase (AstC) from *Escherichia coli*. The B Subunit of apo AstC (PDB ID: 4ADEb) disordered in the α1-α2, α6-α7 helix domain and linker loop on the dimer interface.

The conformational stability and the flexibility of proteins is known to be altered upon ligand binding. The change in conformational stability or flexibility in functional regions of enzyme that occurs upon ligand binding depends on the protein type, and it could affect substrate specificity as well as enzyme activity. Interestingly, other apo states of transaminases also have rearranged (or disordered) domains around the catalytic region, similar to that found here for apo Vfat. After performing a structural homology search with the Dali sever^27^, we found three class-III transaminases which also exhibit a loss of stability, or structural change upon loss of the PLP cofactor (Table 2). One of these is a class-III transaminase from *Bacillus subtilis* (Mtb DAPAS), whose structures were resolved in both the PLP bound form and the apo form^28^. The overall structure of the PLP-bound Mtb DAPAS (PDB ID: 3DU4) showed a similar structure to PLP/PMP-bound Vfat. On the other hand, apo Mtb DAPAS (PDB ID: 3DRD) lost conformational stability in the α6-α7 helix domain (residues at 144–172), indicating that the conformational stability of the catalytic region is reduced when the cofactor is absent (Fig. 7A). Another case was the catabolic N-succinylornithine transaminase (AstC) from *Escherichia coli*. Subunit B of apo AstC (PDB ID: 4ADEb) lost conformational stability in the α1-α2, α6-α7 helix domain, and the linker loop at the dimer interface, whereas subunit A (PDB ID: 4ADEa) had a similar overall structure to PLP-bound AstC (PDB ID: 4ADE), having a stable structure in all these regions (Fig. 7B)^29^. The structural differences between the two subunits of apo AstC indicate that the stability of apo transaminase could be diverse in one asymmetric unit, which is the same as the case seen here for apo Vfat. The last example was a transaminase from *Chromobacterium violaceum* (Cv-ωTA) which showed ordered- or disordered structures in different regions of the two subunits from one asymmetric unit^25^. In apo Cv-ωTA and PLP Cv-ωTA (PDB ID: 4A6U and 4A6T, respectively), ordered- or disordered regions were similar to Mtb DAPAS and AstC. However, two different forms of Cv-ωTA (PDB ID: 4A72), apo dimer and PLP-bound dimer, were found in one asymmetric unit^25^. Interestingly, the apo Cv-ωTA dimer (subunit A and B) showed similar patterns in the disordered region to our apo Vfat. In particular, one subunit showed a disordered region in the α1-α2 helix domain, while other subunit had a structurally stable folding of the α1 helix but lost its conformation in the α6-α7 helix domain. Because two more structures of apo form of class III transaminases (PDB ID: 3HMU and 3N5M) are available^30,31^, we compared our structure to them. Although these structures are apo-form, they contain sulfate ion at the PLP binding site. Interestingly, sulfate ion binds to active site K285 reside, which is important for the stability of active site, and contributes to the stability of α1- α2 helixes and α6-α7 (Supplemtary Fig. S5). Based on the crystal structure of the apo state of Vfat, and comparison with other apo proteins, we conclude that the absence of a cofactor can affect the conformational stability and flexibility of the dimer interface, including the catalytic region. Dissociation of cofactor followed by monomerization and structural instability has been also suggested by studying of amine transaminase^21–23^. Our apo Vfat structure is the first in which the linker loops at dimer interface lose their balance. With the recently suggested activation mechanism of transaminase by PLP-substrate dissociation, enzyme stability alternation, and stoichiometric changes^21–23^, our findings with structure of dimeric, PLP-free form of apo Vfat provides a better understanding of structural alteration and instability of transaminase without cofactor.

**Table 2:** Structural similarity search using DALI.

	PDBid:PLP	PDBid:Apo	Reference
*Bacillus subtilis*	3DU4	3DRD	^28^
*Escherichia coli*	4ADB	4ADE	^29^
*Chromobacterium violaceum*	4A6T	4A6U	^25^

## Methods

### Protein expression and purification

The expression and purification methods used in this study have been described elsewhere in detail^32^. In summary, the coding region of Vfat was amplified by PCR and inserted into the vector pET24ma. The pET24ma vector (constructed by Dr. David Sourdive, Pasteur Institute, France) contains a p15A replication origin. The plasmid was transformed into BL21 (DE3) *E. coli* competent cells, and protein expression was induced by treating bacterial cultures with 0.5 mM isopropyl β-D-thiogalactopyranoside (IPTG) overnight at 20 °C. The bacterial cells expressing Vfat were pelleted by centrifugation, resuspended, and lysed by sonication. The lysate was then centrifuged, after which the supernatant fractions were applied to a gravity-flow column (Bio-Rad) packed with Ni-NTA affinity resin (Qiagen). The C-terminal His-tagged Vfat was eluted from the column using elution buffer (20 mM Tris buffer pH 7.9, 500 mM NaCl and 250 mM imidazole). The elution fractions were collected, combined and concentrated to 20–25 mg mL^−1^ using a concentration kit (Millipore). The concentrated protein was then applied to a Superdex 200 gel filtration column 10/30 (GE healthcare) that had been pre-equilibrated with a solution of 20 mM Tris at pH 8.0 and 150 mM NaCl. The protein that eluted at around 14.5 mL upon gel-filtration chromatography was collected and concentrated to 10~12 mg/mL for crystallization.

### MALS

The absolute molar mass of Vfat was determined by multi angle light scattering (MALS). The target protein was loaded onto a Superdex 200 HR 10/30 gel-filtration column (GE Healthcare) that had been pre-equilibrated in a buffer containing 20 mM Tris-HCl pH 8.0 and 150 mM NaCl. The AKTA chromatography system (GE Healthcare) was coupled to a MALS detector (miniDAWM TREOS) and a refractive index detector (Optilab DSP) (Wyatt Technology).

### Crystallization and data collection

Crystallization was conducted at 20 °C by the hanging drop vapor-diffusion method using various screening kits. Initial crystals were grown on the plates by equilibrating a mixture containing 1 μl of protein solution (10–12 mg/ml protein in 20 mM Tris at pH 8.0, 150 mM NaCl) and 1 μl of a reservoir solution from number 3 of Wizard III (20% PEG 3350 and 0.2 M Magnesium formate) against 0.4 ml of reservoir solution. Crystallization was further optimized by searching over a range of concentrations of protein, PEG 3350, magnesium formate. Crystals appeared within two days and grew to a maximum dimension of 0.2 × 0.2 × 0.1 mm in the presence of 22% PEG 3350, 0.4 M magnesium formate and 0.1 M sodium citrate pH 6.2. A 2.0 Å native dataset was collected at the BL-4A beamline at Pohang Accelerator Laboratory (PAL), Republic of Korea. Data processing and scaling was carried out using HKL2000.

### Structure determination and analysis

The structure was determined by the molecular replacement phasing method using Phaser^33^. The previously solved putative aminotransferase structure (PDB code: 3GJU), which shares 33% sequence identity with that of the Vfat, was used as a search model. Model building and refinement were performed in COOT^34^ and Refmac5^35^, respectively. Water molecules were added automatically with the ARP/wARP function in Refmac5 and then examined manually for reasonable hydrogen bonding possibilities. The quality of the model was checked using PROCHECK^36^ and was found to be reasonable. A total of 95.47% of the residues were shown to be located in the most favorable region, while the additional 4.53% were in allowed regions of the Ramachandran plot. The data collection and refinement statistics are summarized in Table 1. Ribbon diagrams and molecular surface representations were generated using Pymol^37^.

### Protein Data Bank accession codes

Coordinates and structural factors were deposited in the Protein Data Bank under PDB ID code 5ZTX.

## Electronic supplementary material

Supplementary figures
