# Supplementary material for: Structural dynamics of the transaminase active site revealed by the crystal structure of a co-factor free omega-transaminase from Vibrio fluvialis JS17
Source: Sci Rep. 2018 Jul 30;8:11454. doi: 10.1038/s41598-018-29846-0 (PMC6065307; doi:10.1038/s41598-018-29846-0)
Supplement: Supplementary file 1 — Supplementary figures [file 41598_2018_29846_MOESM1_ESM.docx]

**MICROBIOLOGY, BIOCHEMISTRY, STRUCTURAL BIOLOGY**

^*^Hyun Ho Park, Phone: +82-2-820-5930. Fax: +82-53-810-4516. E-mail: xrayleox@cau.ac.kr

**Structural dynamics of the transaminase active site revealed by the crystal structure of a co-factor free omega-transaminase from *Vibrio fluvialis* JS17**

Young-Cheul Shin^a^, Hyungdon Yun^b^, and Hyun Ho Park^c,*^

^a^Department of Cell Biology, Harvard Medical School, Boston, Massachusetts 02115, USA

^b^Department of Bioscience & Biotechnology, Konkuk University, Seoul 143-701, Republic of Korea

^c^College of Pharmacy, Chung-Ang University, Seoul 06974, Republic of Korea

Supplementary Figure S1


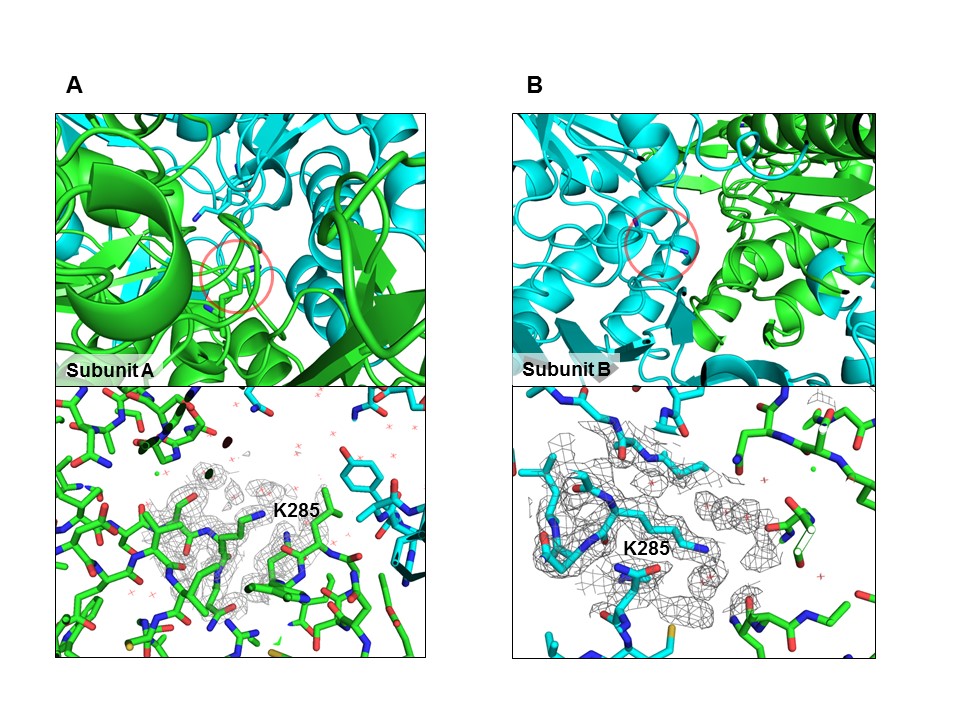


Figure S1. Absence of the co-factor in the active site. Subunit A (A) and subunit B (B) did not contain a cofactor such as PMP or PLP. The red-circle in the upper panel indicates the active site K285 residue which is important for co-factor binding. The lower panel shows the 2Fo-Fc electron density map contoured at 1σ around K285.

Supplementary Figure S2


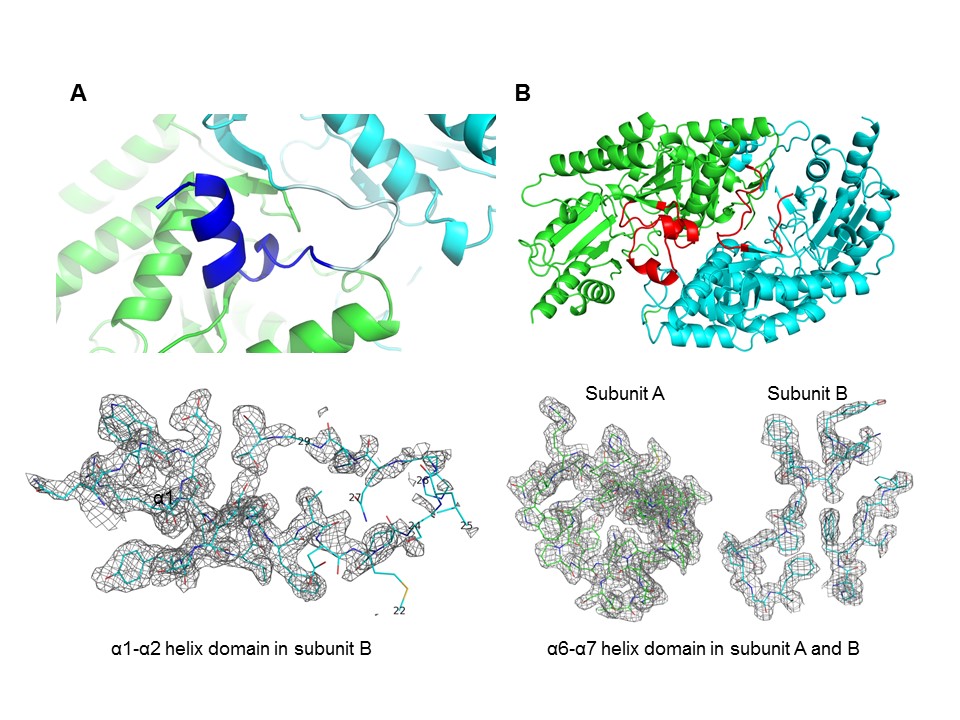


Figure S2. Absence or presence of the α1-α2 helix domain (N-terminal helix domain) and the α6-α7 helix domain. A Presence of the α1-α2 helix domain in subunit B (but not in subunit A). The domain in subunit B is colored dark blue. The lower panel shows the poorly visible 2Fo-Fc electron density map contoured at 1σ around the α1-α2 helix domain of subunit B. B. Presence of the α6-α7 helix domain in subunit A, and absence of the α6-α7 helix domain in subunit B. The lower panel shows the 2Fo-Fc electron density map contoured at 1σ around the α6-α7 helix domain of subunit A and subunit B.

Supplementary Figure S3


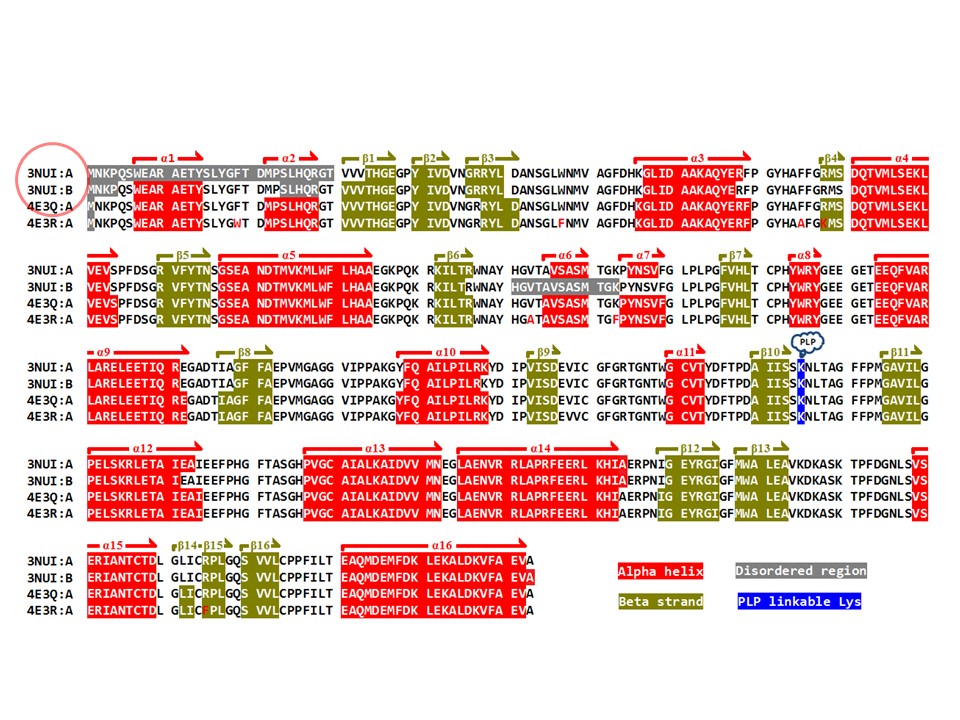


Figure S3. Amino acid sequence and structural alignment of various forms of Vfat. 3NUI:A and 3NUIB are the apo state of Vfat. 4E3Q:A and 4E3R:A are the PMP bound form and PLP bound form of Vfat, respectively. Based on the PDB data, the secondary structures were highlighted red (helix) and lemon (strand). The grey colored sequences indicate the disordered region in the final models. The lysine at residue 285, which is the most critical residue in all transaminases, is colored blue.

Supplementary Figure S4


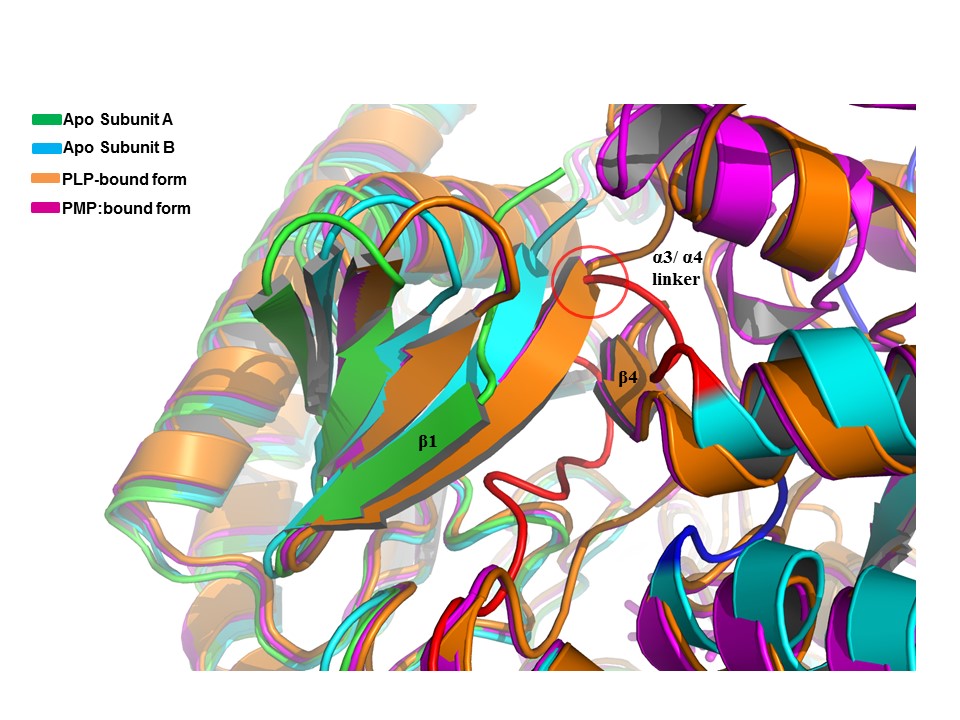


Figure S4. Shortened structure of the β1 strand in subunit A. The relatively short β1 strand in subunit A (green) is shown. The red circle indicates the longer β1 strand. The α3-α4 liker in subunit B is shown in red.

Supplementary Figure S5


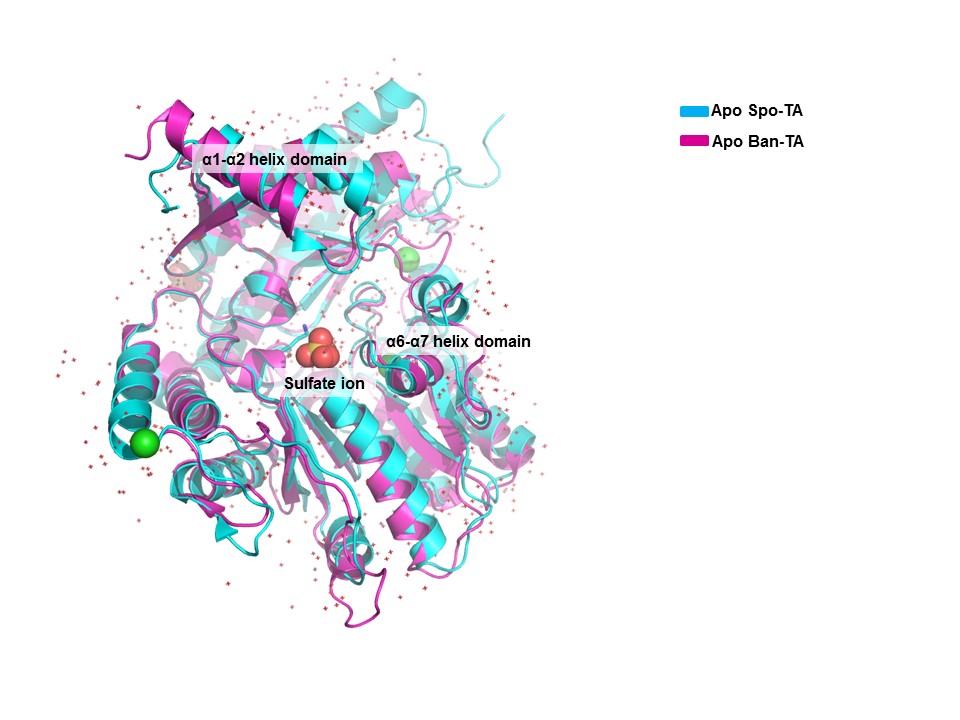


FIGURE S5. Sulfate ion –mediated structural stability of the active site of other transaminases. A. The structures of apo form of Spo-TA (PDB ID: 3HMU) and apo form of Ban-TA (PDB ID: 3N5M). Spo-TA and Ban-TA is class III transaminase from *Silicibacter pomeroyi* and *Bacillus anthracis*, respectively.
